# Log P Determines Licorice Flavonoids Release Behaviors and Classification from CARBOMER Cross-Linked Hydrogel

**DOI:** 10.3390/pharmaceutics14071333

**Published:** 2022-06-24

**Authors:** Zhuxian Wang, Yi Hu, Yaqi Xue, Zhaoming Zhu, Yufan Wu, Quanfu Zeng, Yuan Wang, Chunyan Shen, Qun Shen, Cuiping Jiang, Li Liu, Hongxia Zhu, Qiang Liu

**Affiliations:** School of Traditional Chinese Medicine, Southern Medical University, Guangzhou 510515, China; wangzhuxian8998@163.com (Z.W.); huyi12110357@smu.edu.cn (Y.H.); xyq1997@smu.edu.cn (Y.X.); zmnf1988@smu.edu.cn (Z.Z.); 3161008010@smu.edu.cn (Y.W.); 22020286@smu.edu.cn (Q.Z.); 521wl@smu.edu.cn (Y.W.); shenchunyan@smu.edu.cn (C.S.); sq@smu.edu.cn (Q.S.); jxiaqing126@smu.edu.cn (C.J.); 3188010173@i.smu.edu.cn (L.L.)

**Keywords:** carbomer hydrogel, intermolecular interaction, drug release, quantitative structure–activity relationship, log P, licorice flavonoids

## Abstract

The dynamic drug release mechanisms from Carbomer 940 (CP) hydrogels have not been systematically explored elsewhere. This study aimed to investigate the quantitative structure−activity relationship of licorice flavonoids (LFs) compounds on their drug release from CP hydrogels based on LFs-CP interactions and drug solubility in the release medium. Ten LFs-CP hydrogels were formulated, and their in vitro release study was conducted. The intermolecular forces of LFs-CP systems were characterized by FTIR, molecular docking and molecular dynamic simulation. Ten LFs compounds were classified into I (high-release capability) LFs and II (low-release capability) LFs according to the different negative correlations between drug release percent at 48 h and intermolecular forces of drugs-CP, respectively. Moreover, high-release LFs possessed significantly lower log P and higher drug solubility in the release medium than low-release LFs. All I LFs release behaviors best followed the first-order equation, while II LFs release characteristics best fitted the zero-order equation except for isoliquiritigenin. Log P mainly affect the hydrogel relaxation process for I drugs release and the drug diffusion process for II drugs release. Higher log P values for LFs resulted in higher intermolecular strength for I drugs-CP systems and lower drug solubility in the release medium for II drugs, which hindered drug release. Hydrophobic association forces in drug-CP hydrogel played a more and more dominant role in hindering I LFs release with increasing release time. On the other hand, lower drug solubility in the release medium restricted II LFs release, and the dominant role of drug solubility in the release medium increased in 24 h followed by a significant decline after 36 h. Collectively, log P of LFs served as a bridge to determine LFs compound release behaviors and classification from CP hydrogels, which provided guidelines for reasonable design of LFs hydrogels in pharmaceutical topical formulations.

## 1. Introduction

Hydrogels are used extensively in topical and transdermal drug delivery systems, Licorice flavonoids (LFs) are a class of polyphenolic compounds having a basic C_6_-C_3_-C_6_ skeleton derived from licorice, and are classified as flavones, flavonols, chalcones, isoflavones, etc. Their lipophilic nature and poor solubility lead to a limited oral bioavailability, and the topical route is the best administration route for LFs. The antioxidant, photo-protective, anti-inflammatory and anti-aging activities of LFs enable them to be applied ubiquitously in skin disorders with minimum side effects [1,2,3]. Liquiritin (LiQ) [4] and isoliquiritin (IsL) [5] are effective in atopic dermatitis, while isoliquiritigenin (IsLT) [6] and licochalcone B (LB) [7] play a vital role in suppressing melanoma cells in the skin. Glabridin (Gla 1) [8] and licochalcone A (LA) [9,10] possess remarkable anti-melanogenic effect as we previously found, and liquiritigenin (LiQT) [11] is extensively used in anti-aging cosmetic formulations. The skin protective activities of LFs compounds provide them with great potential for application in topical formulations such as hydrogels [12]. Drug release [13] is the first important step of drug permeation from hydrogel, followed by percutaneous absorption [14] into skin. However, these compounds are distinct in physicochemical parameters including molecular weight, log P, polarizability, polar surface area, etc., which could be the main reason for the discrepancy in drug release from hydrogel polymers. Still, few studies have focused on LFs release capability from polymers as a function of physicochemical properties of these drugs.

As one of the successfully controlled release vehicles, Carbomer 940 (CP) hydrogel is still known as a thickener in topical preparations due to its moderate rheological properties and high storage stability [15,16]. Drug release from hydrogels involves the hybridization of hydrogel relaxation (or hydrogel degradation) and drug diffusion; usually, more than one mechanism controls drug release in a release system. Drug diffusion is often accompanied by drug relaxation and might be a limiting step at different periods of drug release [17,18]. Intermolecular strength and viscosity and loss modulus (G″) of the hydrogel influence the hydrogel relaxation [19,20,21], while drug diffusion primarily depends on the mesh size of the hydrogels [22,23], relative partitioning of solubilized drugs between the hydrogel and the release medium, drug solubility in the release medium [24], etc. Previous studies reported that the stronger the interaction of hydrogen bonds or van der Waals forces between the pressure-sensitive adhesive (PSA) matrix and drug components, the lower the drug release percent and rate were [13]. The high viscosity, G″, and small mesh size of the hydrogel also hindered the drug release [19,25]. In some hydrogel systems, intermolecular interaction was the dominant factor hindering drug release, while drug solubility in the release medium become the decisive factor controlling drug release in other hydrogel systems [21].

Until now, how LFs compounds release from CP hydrogels and the dynamic molecular mechanisms involved in a release system at different stages have not been systematically explored elsewhere. LFs compounds, with various structures, showed different affinities for CP, resulting in different interactions with CP polymer, which formed hydrogels with different viscosity properties. The carboxyl (–COOH) and methylene (–CH_2_) group of the Carbomer chain and the hydroxyl (–OH), carbonyl (C=O) and hydrophobic groups of the LFs compounds were the functional groups that may potentially be involved in LFs-CP interactions [26]. Therefore, the specific interaction between LFs compounds and CP and their structure−release activity relationships in hydrogels should be illustrated, which will shed new light on the possible release mechanisms of LFs ingredients from hydrogels and provided guidance for practical topical application of flavonoids compounds.

Therefore, the study aimed to investigate the quantitative structure−activity relationships of LFs compounds with their drug release capability at different times and further summarize the potential release mechanisms of LFs ingredients in hydrogels. A series of LFs compounds including LiQ, IsL, LiQT, IsLT, Gla 1, LA, Lico A, LB, ReT and Gla 2 were selected for model drugs. Their structures and physicochemical parameters are given at Figure S1 and Table 1. Firstly, ten LFs-CP hydrogels were prepared, and their rheological properties and drug solubility in the release medium were characterized. Subsequently, their drug release behavior was assessed by in vitro drug release experiments. The intermolecular forces between different LFs and CP were quantitatively demonstrated by FT-IR, molecular docking and molecular dynamics simulations. Furthermore, the drug release mechanisms were revealed by polarized light microscopy (PLM), scanning electron microscopy (SEM) and X-ray diffraction (XRD). In addition, the correlation between the drug release percent at different times (6,12, 24, 36 or 48 h) and physicochemical parameters of the drugs, drug solubility in the release medium, viscosity and G″, C=O and –OH band displacement values in FTIR analysis and cohesive energy density (CED) were investigated, respectively. The results provided insight into the structural characteristics of LFs in CP hydrogel release, and provide information for topical and transdermal applications of flavonoids with specific structures to achieve high release.

## 2. Materials and Methods

### 2.1. Materials

Liquiritin, isoliquiritin, liquiritigenin, isoliquiritigenin, glabridin, licochalcone A, licoflavone A and glabrone (purities > 98%) were purchased from Nanjing Spring & Autumn Biological Engineering Co., Ltd. (Nanjing, China). Licochalcone B and retrochalcone (purities > 98%) were supplied by Chengdu Chroma-Biotechnology Co., Ltd. (Chengdu, China). Carbomer 940 (molecular weight: 7 × 10^5^–4 × 10^6^) was from Shanghai Macklin Biochemical Co., Ltd. (Shanghai, China). Phosphate-buffered saline (PBS, pH = 7.2) and sodium hydroxide were purchased from Shanghai Macklin Biochemical Technology Co., Ltd. Polyethylene glycol 400 (PEG 400), and cellophane membranes were obtained from Beijing Solarbio technology Co., Ltd. (Beijing, China). Acetonitrile and methanol for HPLC analysis was obtained from Merck Co., Ltd (Darmstadt, Germany). All other reagents were analytical grade.

### 2.2. Preparation of Hydrogels

Ten kinds of LFs-CP hydrogels (drug loading: 5%, *w*/*w*) were formulated as follows: LFs monomer compounds in ethanol solution were mixed with Carbomer 940, which was fully swelled in water beforehand, and the mixture was stirred until homogeneous. Finally, the pH was adjusted to 5 with NaOH solution [21,27,28]. Hydrogel films were obtained by freeze-dried technique until there was no residual water in the films before analysis.

### 2.3. Drug Solubility in the Release Medium

Briefly, an excess of compounds was added into the PBS/PEG400 (*v*/*v*, 80/20), followed by treating with ultrasound (Ultrasonic cleaner, KQ-100, China) at 100 W for 30 min at 25 °C. The 10 LFs compounds showed great stability after ultrasound in this mode. Subsequently, the solution was centrifuged and filtered with a 0.22 μm microporous membrane. The samples were then analyzed by HPLC (Agilent 1260, USA) equipped with C18 column (5 μm, 4.6 × 250 mm) and DAD detector. Moreover, the standard curve of each LFs compound based on the peak areas in the Agilent Technologies offline program was utilized to analyze the drug solubility. The HPLC methods were as follows. The mobile phase consisted of combinations of A (acetonitrile), B (methanol)and C (0.1% phosphate in water, *v*/*v*) at a flow rate of 1.0 mL/min with an elution gradient as follows: 0min, 15% A and 10% B; 5 min, 22% A and 12% B; 10 min, 32% A and 14% B; 20 min, 45% A and 20% B; 20–30 min, 45% A and 20% B; 40 min, 15% A and 10% B. The detection wavelength was set at 300 nm, and the injection volume was 20 μL.

### 2.4. Rheological Study

The rheological study was conducted using an MARS iQ Air+P35 rheometer (Thermo Scientific HAAKE, New York, NY, USA). Flow properties of the hydrogels were obtained from continuous shear flow tests with the shear rates at 0–120 s^−1^ for 120 s. The G″ value was measured by frequency sweep tests ranging from 0.1% to 100% strain at constant frequency [21,27,29].

### 2.5. XRD and PLM

X-ray powder diffraction (XRD) was used to determine the proportion of crystalline in different hydrogels. Patterns of dry samples were obtained by an X-ray diffractometer (SmartLab 3KW, Rigaku, Japan) using a Cu Kα radiation source with intensity and voltage at 30 mA and 40 kV. The angular range of data acquisition was 5–60° 2θ with a scan rate of 10°/min. The crystallinity index (the percentage of crystalline) was calculated as follows:

(1) $$\mathrm{Crystallinity}\;\mathrm{index}=\frac{\mathrm{Peak}\;\mathrm{intensity}}{\mathrm{Total}\;\mathrm{diffraction}\;\mathrm{intensity}\;}\times 100\%$$

PLM measurement was conducted to observe the crystalline in hydrogel film, which served as a supplementary tool for XRD results. The dry samples were viewed under a Nikon polarized optical microscope (Eclipse LV100N POL, Tokyo, Japan), and QImaging software (Nis-Elements F) was utilized to view the images with a first-order compensator at 100× magnification.

### 2.6. Attenuated Total Reflection FT-IR (ATR-FT-IR)

The interactions between LFs and CP matrix were confirmed by the movements of the characteristic bands on a Nicolet iS50 FT-IR spectrometer (American Thermos, New York, NY, USA). The spectra of freeze-dried drug-loaded hydrogels were obtained through 32 scans in the 4000–500 cm^−1^ at resolution 2.

### 2.7. Raman Spectroscopy

The potential interactions between different LFs compounds and carbomer matrix in the hydrogel films were evaluated by a Raman spectrometer (Renishaw RM2000, London, England). The samples were measured at 25 °C using a 785 nm laser source with 500 mW power.

### 2.8. SEM

The dried hydrogel samples were cut into small squares, fixed on aluminum stubs and coated with evaporated gold. SEM (FEI Quanta 400 FEG, American FEI) was used to observe the surface morphology including the size of mesh pores of the hydrogels at an acceleration voltage of 20 KV. The average pore size of hydrogel was recorded by assuming 5 imaging taken for each sample.

### 2.9. In Vitro Release of the Hydrogels

Drug release experiments were conducted using horizontal diffusion cell methods [21,30,31,32]. The hydrogels (0.3 g) were pasted onto cellophane membranes, respectively, which were clamped between the donors and receptors compartment of the horizontal diffusion cells (TP-6, Tianjin JingTuo Instrument Technology Co., Ltd., Tianjin, China). Moreover, the effective diffusional area was 1.54 cm^2^, and the volume was 15 mL. The cellophane membrane was immersed in PEG 400/PBS (*v*/*v*, 20/80) (to maintain sink condition) at 350 rpm stirring and 32 °C (the temperature was similar to human skin surface temperature) for 48 h. A 1.0 mL sample was collected at 0.25, 0.5, 1, 2, 3, 4, 6, 8, 10, 12, 24, 36 and 48 h and then replaced by the same volume of fresh medium. Drug concentration was determined by HPLC (Agilent 1260, Santa Clara, CA, USA) as described before. 

### 2.10. Molecular Interaction Study: Molecular Docking

Materials Studio version 8.0 (Accelrys, San Diego, CA, USA) was used for molecular docking, which was conducted to elucidate the interaction strength between drugs and CP and determine the existence form of interaction forces. The structures of 10 LFs compounds and CP were obtained from the PubChem database and subjected to geometry optimization using the Forcite module based on a smart algorithm. After that, the blend module was utilized to calculate the interaction parameters (χ). The best docking structures were obtained.

### 2.11. Molecular Dynamic Simulation

In molecular dynamic study, an amorphous cell module was used to construct a simulated system consisting of optimized LFs compounds and CP according to the actual proportions. Subsequently, the LFs-CP systems were further optimized by Forcite modules in the Compass II force field. After that, runs of 50 ps at 298 K in the (canonical ensemble) NVT and 100 ps at 305 K and 101.325 Kpa in the (constant-pressure, constant-temperature) NPT on each system were performed to obtain equilibrium structures. The cohesion energy density (CED) of the LFs-CP was calculated to characterize the interaction force between drug and CP [13,33].

### 2.12. Correlation Analysis 

Firstly, a linear regression analysis was performed to investigate the correlation between the drug release amounts at 6, 12, 24, 36 and 48 h and C=O and –OH displacement values in FTIR, χ and CED between drugs and CP using SPSS 21.0 software, respectively. The drug release amounts at different time points as a function of physicochemical parameters (log P, polarizability and polar surface area) of the drugs, drug solubility in the PBS/PEG400 (*v*/*v*, 80/20), and rheological parameters (the zero-shear viscosity and G″) were demonstrated, respectively. 

### 2.13. Statistical Analysis

All data were analyzed using ANOVA and the Student’s *t*-test using SPSS 21.0 software (SPSS, Chicago, IL, USA). A probability of *p* < 0.05 was taken as significant difference. Correlation analysis revealed a significant correlation between the dependent variable and independent variables when |r| > 0.95. Moreover, it was considered as highly correlated when |r| ≥ 0.80.

## 3. Results

### 3.1. Characterization of the LFs-CP Hydrogels

**Figure 1:**
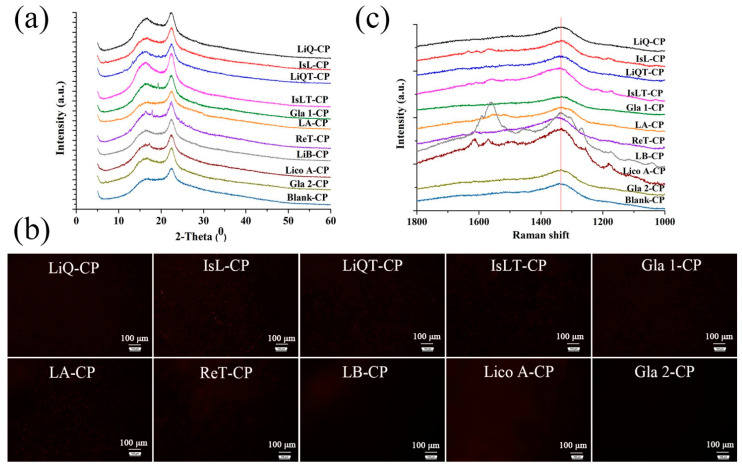
Characterization of LFs-CP hydrogel. (**a**) X-ray powder diffractograms of ten LFs-CP hydrogels; (**b**) PLM images of LFs-CP films (The black represented that the drug was molecularly dispersed in the hydrogel films while red represented the crystals. Bar = 100 μm); (**c**) Raman spectra of different hydrogels.

Ten kinds of LFs-CP hydrogels were formulated by stirring methods to ensure that the compounds were completely dispersed in the hydrogels, and most importantly, LFs-CP interactions were formed. XRD (Figure 1a) and PLM (Figure 1b) technologies were applied to detect the crystals in the dry hydrogel films. The results demonstrated that most LFs compounds were molecularly dispersed in the hydrogel except for the Gla 1, ReT and Lico A, whose crystallinity indexes were 2.19%, 3.12% and 1.04%, respectively, suggesting the amorphous nature of LFs compounds in the hydrogels, and good compatibility was present between LFs compounds and CP matrix. This also laid a foundation for the formation of intermolecular forces between the drug and the CP matrix [34].

The water contents in these freeze-dried materials had been tested to ensure that there was no residual water, which could impact the appearance and shifts of –OH bands in the FTIR and Raman analysis. Moreover, the change in LFs compounds’ –OH and C=O band areas and shifts was not claimed after mixing with CP matrix due to the low content of LFs compounds (5%, *w*/*w*) in the hydrogel. The Raman spectra (Figure 1c) showed that different movements of bands at approximately 1300 cm^−1^ representing the –OH group were obtained in CP hydrogel when different LFs were added. Moreover, the –OH vibration band area in LFs-CP was larger (IsLT, ReT, LB, Lico A and Gla 2) or smaller (IsL, LiQ, LiQT, Gla 1 and LA) compared to that in blank CP, indicating the formation of intermolecular forces. The results of quantitative analysis of the intermolecular forces of LFs-CP binary systems are shown as follows. 

### 3.2. In Vitro Release of LFs-CP Hydrogels

**Figure 2:**
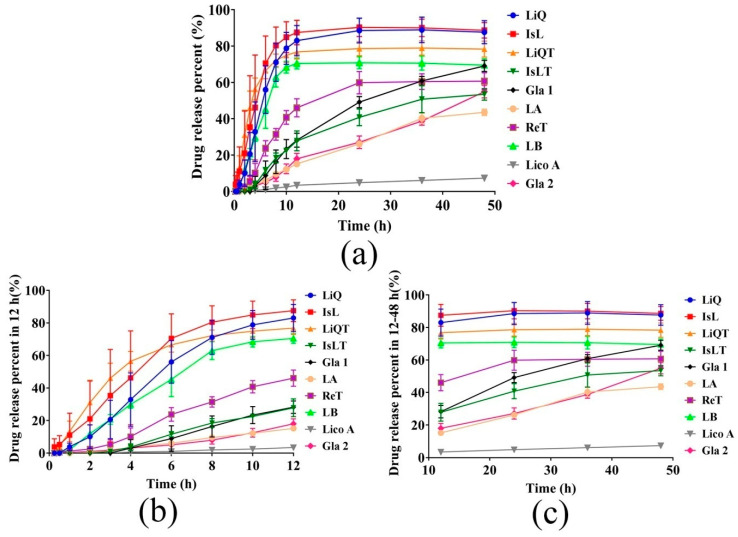
(**a**) In vitro drug release profiles of ten LFs-CP hydrogels in 48 h (n = 3). (**b**) In vitro drug release profiles of ten LFs-CP hydrogels in the first 12 h (n = 3). (**c**) In vitro drug release profiles of ten LFs-CP hydrogels in 12–48 h (n = 3).

The results (Figure 2) showed that different LFs compounds displayed different release percent from CP matrix in 48 h, and the release percent was rank-ordered as IsL (88.67%) > LiQ > LiQT > LB > Gla 1 > ReT > Gla 2 > IsLT > LA > Lico A (7.35%). We can see that the PEG 400/PBS (*v*/*v*, 20/80) as the release medium met the sink condition for all drugs’ release. IsL, LiQ, LiQT, LB and ReT reached their maximum release at 12 h. However, the released amounts of Gla 1, Gla 2, IsLT, LA and Lico A were enhanced as the time increased to 48 h. LiQ and IsL, and LiQT and IsLT, are isomers. However, the release percent of LiQT was significantly higher than that of IsLT, while LiQ and IsL displayed similar release capability. Moreover, the release behaviors of IsL, LiQ, LiQT, LB and RET from CP hydrogel best followed the first-order equation, while the release behaviors of Gla 1, Gla 2, LA and Lico A best fitted the zero-order equation, and IsLT best followed the Higuchi equation (Table 2 and Table S3). Next, FTIR, rheological analysis, SEM, molecular docking and molecular dynamic simulations were performed to systematically demonstrate the influencing effects on drug release.

### 3.3. LFs Release Classification According to FTIR Analysis and Drug Release Percent

**Figure 3:**
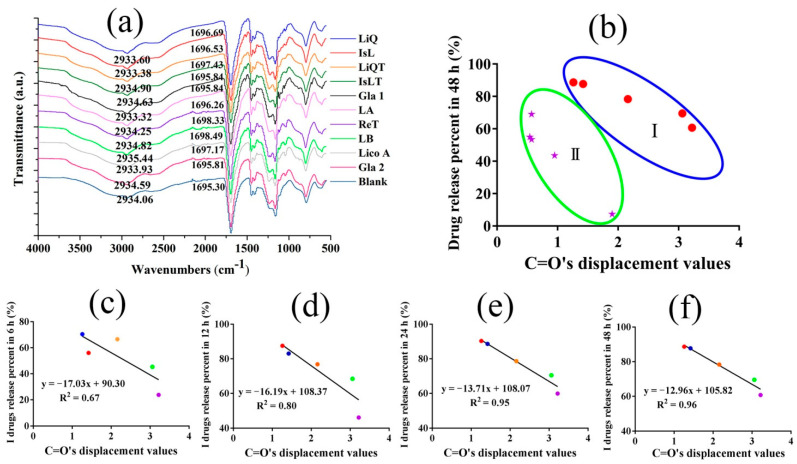
(**a**) FTIR spectra of LFs-CP hydrogels. (**b**) The linear correlation of the ten kinds of LFs release percent in 48 h and C=O band displacement values (The red dots represented I drugs while purple stars represented II drugs). The relationships between I drug (high-release LFs) release in 6 h (**c**), 12 h (**d**), 24 h (**e**) and 48 h (**f**) and C=O band displacement values in FTIR. (The red, blue, yellow, green and purple dots represented IsL, LiQ, LiQT, LB and ReT respectively).

FTIR was carried out to reveal the potential interaction between different LFs and CP in the freeze-dried samples. In the blank CP (Figure 3a), the characteristic band at 2934.06 cm^−1^ represented –OH stretching vibration, while the band at 1695.30 cm^−1^ was attributed to C=O stretching of the CP. Upon mixing with LB and ReT, the C=O band of CP had the highest red shift, which moved to 1698.33 cm^−1^ and 1698.49 cm^−1^, respectively. However, the C=O band only moved to 1695.84 cm^−1^, 1695.84 cm^−1^ and 1695.81 cm^−1^ after IsLT, Gla 1 and Gla 2 were added, respectively. The addition of other LFs also induced the movement of C=O bands of CP to different positions. Higher displacement values meant stronger intermolecular forces [34,35]. Next, linear regression of the drug release percent in 48 h and C=O band displacement values was performed to clarify the effect of the intermolecular strength on 10 LFs’ release, and we found that no linear correlation was present between the two (Figure 3b). However, there was a good negative linear correlation (R^2^ = 0.96 and R^2^ = 0.93) between the two, respectively, when we classified these LFs into two categories: I (IsL, LiQ, LiQT, LB and ReT) and II (Gla 1, Gla 2, IsLT, LA and Lico A) (Figure 3f and Figure S2a). In addition, I drug release percent at 6 h, 12 h and 24 h was also negatively correlated with C=O band displacement values, and the correlation coefficients (R^2^ = 0.67, 0.80 and 0.94, respectively) were lower than those at 48 h (Figure 3c–e). The results proved the more and more dominating effect of intermolecular strength controlling I drug release as the release time increased. In contrast, II drug release percent at 12 h and 24 h was not correlated with C=O band displacement values, revealing that intermolecular strength was not a key factor controlling II drug release in the first 24 h.

Similarly, LFs also induced the movement of –OH bands to different positions; however, the displacement values were not as significant as those of the C=O bands. The –OH bands of CP also displayed their highest movement after LB and ReT were added. Interestingly, there was also a negative linear correlation (R^2^ = 0.82 and R^2^ = 0.67) between drug release percent in 48 h and –OH band displacement values only when these drugs were classified into I and II categories, as emphasized before (Figure S2b–d). Both C=O and –OH of the CP, especially the C=O group, were the action sites potentially involving the intermolecular strength forming and controlling LFs release from hydrogel.

More importantly, I drugs possessed significantly higher drug release percent and release rates than those of the II drugs (Figure 2); therefore, the I drugs were defined as the high-release LFs, while II drugs represented the low-release LFs. Taken together, these results indicated that the intermolecular strength had a different decreasing effect on I drug and II drug release, and the decreasing effect was dependent on release time.

### 3.4. Rheological Properties and Drug Release

**Figure 4:**
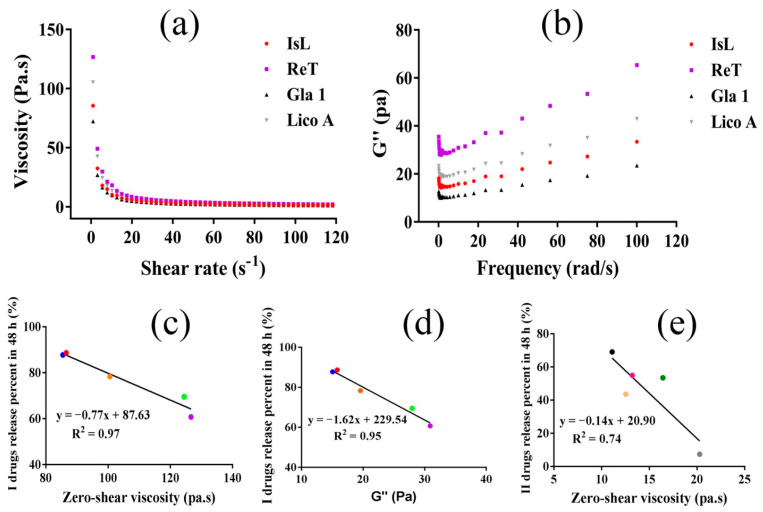
(**a**) Flow characterization of the hydrogels (n = 3, taking IsL-CP, ReT-CP, Gla 1-CP and Lico A-CP as examples). (**b**)Loss modulus (G″) of ten LFs-CP hydrogels (n = 3, taking IsL-CP, ReT-CP, Gla 1-CP and Lico A-CP as examples). The negative linear relationships between I drug release percent in 48 h and zero-shear viscosity (**c**) and G″ value (**d**), respectively. (**e**) II drug release percent in 48 h as a function of G″ value. (The red, blue, yellow, green and purple dots represented IsL, LiQ, LiQT, LB and ReT respectively, while black, pink, dark green, light yellow and gray dots represented Gla 1, Gla 2, IsLT, LA and Lico A respectively).

To confirm the results of FTIR, the zero-shear viscosity and G″ representing the changes in the total intermolecular forces [36,37] of different LFs-CP hydrogels were measured. The LFs-CP hydrogels all presented a decrease in viscosity as the shear rate increased (Figure 4a and Figure S3). For I drugs, ReT-CP hydrogel had the highest zero-shear viscosity and G″ (Figure 4b), while the LiQ and IsL were the lowest, which resulted in the highest IsL release amount and lowest ReT release amount in 48 h. Correspondingly, a negative linear relationship existed between the drug release percent in 48 h and zero-shear viscosity (R^2^ = 0.95) and G″ value (R^2^ = 0.97), respectively (Figure 4c,d). The results further emphasized the dominating role of intermolecular forces hindering I drug release in the whole 48 h release period. For II drugs, Lico A possessed the highest zero-shear viscosity and G″. However, the drug release percent in 48 h only negatively correlated with G″ instead of zero-shear viscosity, and the correlation coefficient only reached 0.74 (Figure 4f). This also proved the weaker role of intermolecular strength in controlling II drug release compared to I drugs.

### 3.5. Drug Solubility in the Release Medium and Drug Release

**Figure 5:**
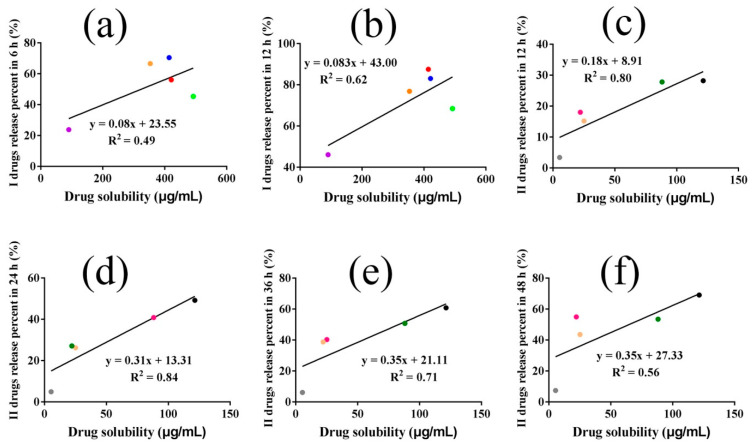
The correlation between I drug release percent in 6 h (**a**), 12 h (**b**) and drug solubility in release medium, respectively; II drug release percent in 12 h (**c**), 24 h (**d**), 36 h (**e**) and 48 h (**f**) as a function of drug solubility in release medium, respectively. (The red, blue, yellow, green and purple dots represented IsL, LiQ, LiQT, LB and ReT respectively, while black, pink, dark green, light yellow and gray dots represented Gla 1, Gla 2, IsLT, LA and Lico A respectively).

To confirm the effect of drug solubility in the medium on drug release, LFs solubility in PBS/PEG400 (*v*/*v*, 80/20) was measured. For I drugs, the drug solubility in the medium followed the order of LB (492.57 μg/mL) > LiQ > IsL > LiQT > ReT (90.49 μg/mL), and for II drugs, Gla 1 (121.37 μg/mL) > IsLT > Gla 2 > LA > Lico A (5.34 μg/mL) (Table 1). It was evident that I drugs possessed significantly higher drug solubility in the release medium than II drugs. The linear relationships between drug release percent in 6, 12, 24, 48 h and drug solubility were demonstrated. We found that for I drugs, the release percent in 6 h, 12 h improved with increasing drug solubility in the release medium, and the correlation coefficients between the two were only 0.49 and 0.62, respectively. However, there was no linear relationship between the two with increasing time (Figure 5a,b). The results demonstrated that drug solubility in the release medium only influenced the first 12 h release of I drugs. For II drugs, the drug release percent values in 12, 24, 36 and 48 h were all positively correlated with drug solubility in the release medium, and the correlation coefficients increased followed by a decrease after 36 h (Figure 5c–f). The results indicated that drug solubility in the release medium affected all stages of the II drug release process, especially in the first 24 h.

### 3.6. The Structure−Activity Relationship of LFs with Their Drug Release from CP Hydrogels

**Figure 6:**
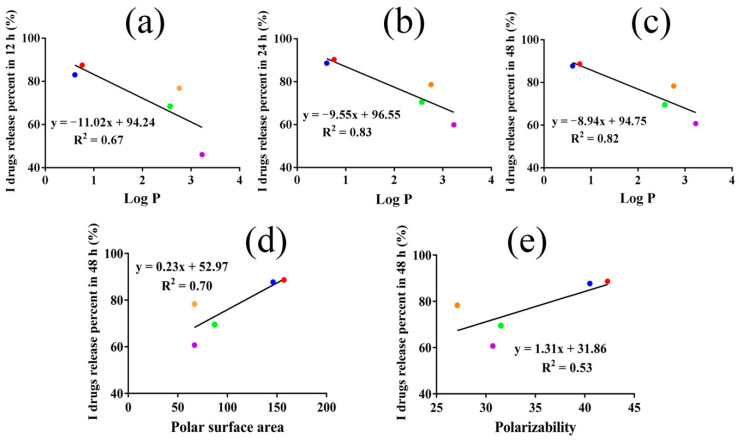
The linear relationship between I drug release percent in 12 h (**a**), 24 h (**b**), 48 h (**c**) and log P of the high-release LFs, respectively. The correlation between I drug release percent in 48 h and polar surface area (**d**) and polarizability (**e**) of the high-release LFs, respectively. (The red, blue, yellow, green and purple dots represented IsL, LiQ, LiQT, LB and ReT respectively).

The structure−release relationships of LFs were then quantitatively analyzed. The 10 LFs compounds selected for analysis ranged in their log P from 0.61 to 5.34, polar surface area from 55.8 to 157, and polarizability from 27.1 to 42.3, which provided a basis for quantitative drug release capability analysis. Next, the correlations of drug release percent at different time intervals with the physicochemical parameters of drugs were assessed. We found that the log P of I drugs was significantly lower than that of II drugs. For I drugs, the drug release percent in 12, 24 and 48 h improved as the log P values decreased, and the correlation coefficients were enhanced along with the increase in time (Figure 6a–c). These indicated that log P directly affected drug release and played a more and more significant role in suppressing I drug release from CP hydrogels as time increased. However, there was no correlation between II drug release percent and the log P, demonstrating that log P did not directly influence II drug release from the CP network.

More importantly, all I drug peaks came earlier than those of the II drugs in HPLC analysis (Figure S8), indicating that the polarities of I drugs were significantly higher than those of II drugs. Furthermore, I drug release percent also possessed a positive relationship with the polar surface area in 12 h and 48 h (R^2^=0.56 and 0.70) (Figure 6d and Figure S4a), and with polarizability in 24 h and 48 h (R^2^=0.53 and 0.53) (Figure 6e and Figure S4b).

### 3.7. Morphology of CP Hydrogels and Drug Release

**Figure 7:**
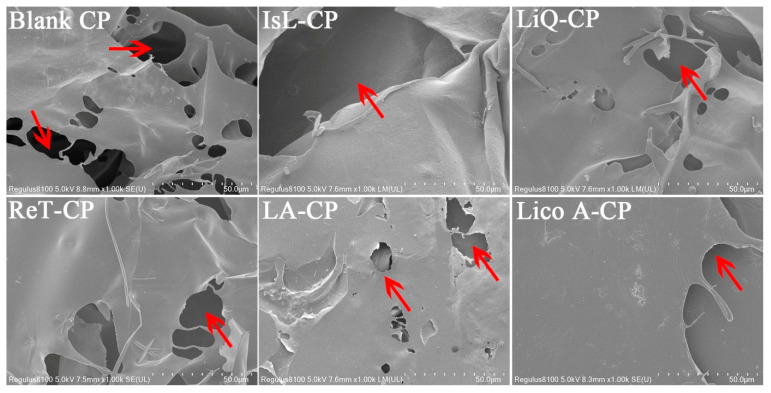
The network structures and pore size distributions of the LFs-CP hydrogels. (Bar = 50 μm, taking blank CP, IsL-CP, LiQ-CP, ReT-CP, LA-CP and Lico A-CP as examples, red arrows represented the mesh pore of the hydrogel).

The micro-morphologies of the freeze-dried LFs-CP hydrogels were shown to possess well-defined network structures by SEM. The larger pore size of the hydrogels was indicative of a decreased cross-linked CP network and a higher drug release capability. It can be seen that the average pore size of I drugs-CP hydrogels was significantly larger than that of II drugs-CP hydrogels (Figure 7, Figure S5 and Table S1), which facilitated IsL, LiQ, LiQT and LB release. Significant amounts of IsLT, LA and Lico A crystals were seen on the surface of hydrogels, which blocked the hydrogel pores and then hindered the drug release. It was reported that the pore size of the hydrogels relied on the size of the ice crystals formed in the freeze-drying treatment of the samples. The greater the degree of CP cross-linking, the more limited the capacity of hydrogels to absorb water [38]. As a result, the size of the ice crystals and hydrogel pores decreased with increases in CP cross-linking.

### 3.8. Molecular Modeling and Molecular Dynamics Simulation

**Figure 8:**
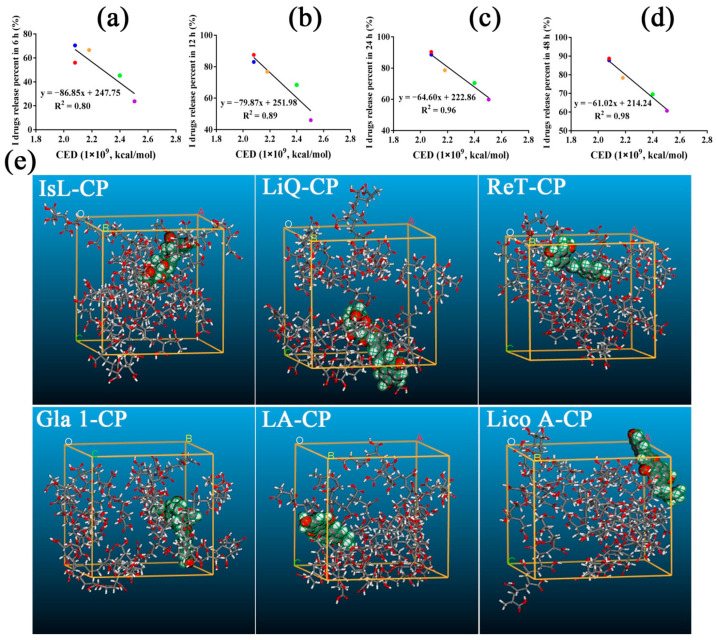
The correlation between I drugs release percent in 6 h (**a**), 12 h (**b**), 24 h (**c**), 48 h (**d**) and CED values of I drugs-CP binary systems, respectively (The red, blue, yellow, green and purple dots represented IsL, LiQ, LiQT, LB and ReT respectively). (**e**) Snapshots of systems at the end of the MD (drug: CPK model, taking IsL-CP, LiQ-CP, ReT-CP, Gla 1-CP, LA-CP and Lico A-CP as examples).

The values of χ and CED were used to calculate the strength of intermolecular interactions between different LFs and CP. The optimized binary associations (Figure S6e) and the snapshots of the hydrogel systems at the end of the MD are displayed (Figure 8e and Figure S7). The closer χ values were to 0 and the higher the CED values, the greater the miscibility and the stronger the intermolecular interactions between the drugs and hydrogel polymer. For I drugs, LiQT-CP possessed the highest χ value, while ReT-CP had the lowest (Table S2). I drug release percent in 12 h, 24 h and 48 h decreased with increasing χ values. For II drugs, Lico A showed the best compatibility with CP, and Gla 1 displayed the worst miscibility. However, there was not a good relationship between drug release percent and χ value. This was because the blending analysis could not represent the actual proportions of the drug and the CP in the hydrogels.

However, for I drugs, ReT-CP systems possessed the highest CED values, which hindered ReT release from the CP hydrogel network. In contrast, IsL possessed the highest release rate. Therefore, a good correlation between the drug release percent in 6, 12, 24, 48 h and CED values was seen, and the correlation coefficients increased as the release time increased (Figure 8a–d). The results were consistent with FTIR analysis. However, there was no correlation between the II drug release percent and CED values.

### 3.9. The Role of Log P in Controlling Drug Release

**Figure 9:**
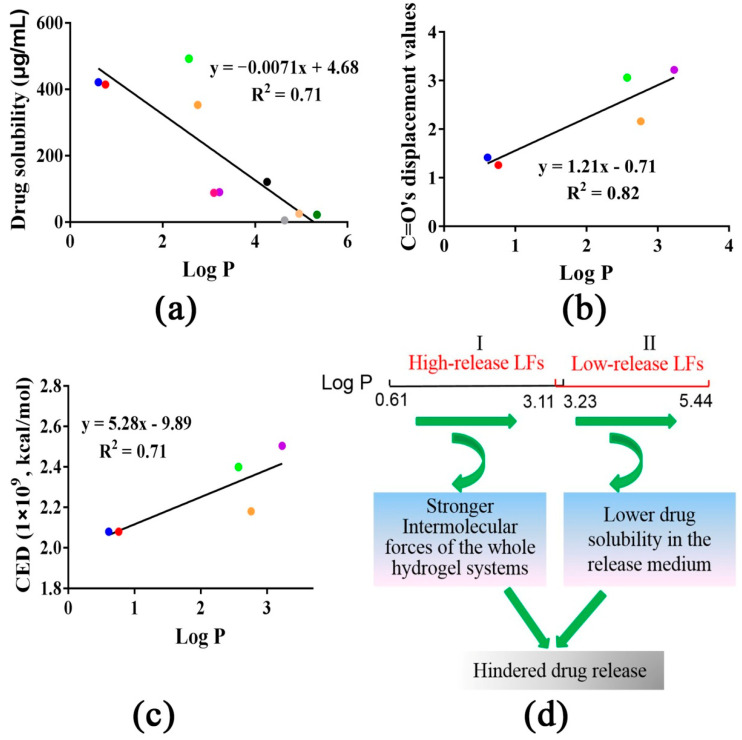
(**a**) The negative correlation between solubility of 10 LFs and log P. (**b**) The positive correlation between C=O band displacement values of I drugs in FTIR analysis and log P. (**c**) The negative correlation between CED values of I drugs and log P. (**d**) Log P of LFs can serve as an important indicator of LFs release classification from CP cross-linked hydrogel. (The red, blue, yellow, green and purple dots represented IsL, LiQ, LiQT, LB and ReT respectively, while black, pink, dark green, light yellow and gray dots represented Gla 1, Gla 2, IsLT, LA and Lico A respectively).

**Figure 10:**
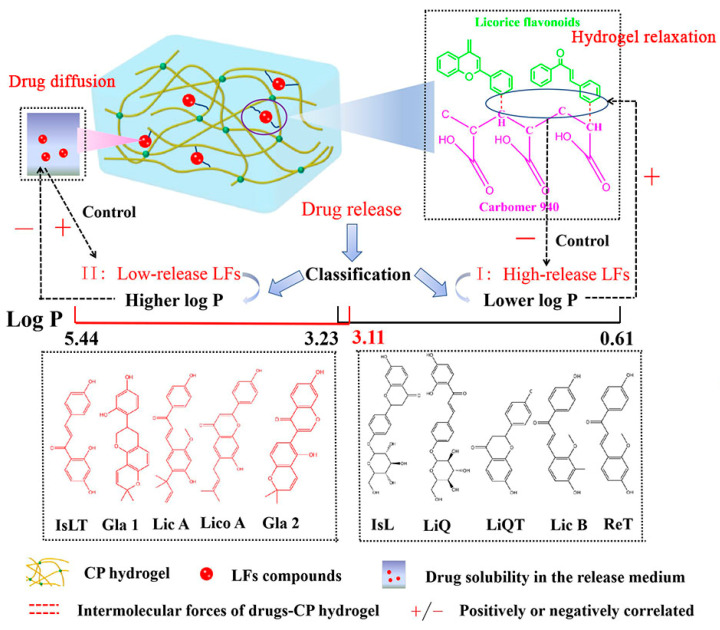
The classification of LFs release from CP hydrogel on the basis of log P, which determined the intermolecular force for I drugs-CP hydrogel and drug solubility in the release medium for II drugs.

As previously demonstrated, the log P of I LFs was significantly lower than that of the II LFs. Therefore, it was expected that log P was a key factor controlling the drug release and determining drug release percent. We can see that a good negative correlation between the drug solubility in the release medium and the log P (R^2^ = 0.71) was obtained (Figure 9a). Thus, for II drugs, the higher log P resulted in lower drug solubility in the release medium, which led to lower drug release percent from the network. In the meantime, for I drugs, both C=O bands’ displacement values and CED values had a good positive correlation with the log P (R^2^ = 0.82 and R^2^ = 0.71), respectively (Figure 9b,c). The results indicated that I drugs with lower log P had better miscibility with CP, thereby resulting in lower C=O band displacement and CED values (stronger LFs-CP interaction), which further contributed to higher drug release percent. Moreover, log P directly affected I drug release and a positive correlation was obtained between the drug release percent in 48 h and log P values (Figure 6c). However, this correlation was not present in the II drugs. The role of log P in determining I drug and II drug release behaviors is shown in Figure 9d and Figure 10.

## 4. Discussion

Although LFs compounds were efficacious in skin ailments and showed their potential in topical hydrogel formulations, no information on the release mechanisms from polymer networks was given before. This study presented comprehensive investigations of LFs compounds’ release from CP hydrogels based on the LFs-CP interaction, hydrogel viscosity and drug solubility in the release medium, and provided a quantitative evaluation of dominant factors controlling drug release at different time points. As a result, the LFs compounds were classified into high-release LFs and low-release LFs according to the log P-release capability relationship analysis (Figure 10).

Firstly, ten LFs compounds were divided into two categories (I and II) due to the different correlations between the drug release percent in 48 h and C=O bands’ displacement values in FTIR analysis (Figure 3b). I and II LFs release percent in 48 h decreased in different trends with increasing intermolecular forces, respectively. Here, the dry CP rather than aqueous CP was utilized to measure the intermolecular strength of whole systems. It was reported that the C=O group displayed a blueshift in CP systems from the hydrated to the dry state, which was indicative of different intermolecular forces. The presence of water in the dried samples reduced the ionic interaction force andincreased the H-bonding between drug and polymers [31,32]. In this work, hydrophobic association forces were the main forces that controlled the drug release, as discussed below; therefore, the dry CP was suitable as an alternative to hydrated CP to measure the hydrogels’ whole energy. Interestingly, we found that I drugs possessed significantly higher release percent values and release rates than the II drugs (Figure 2). Therefore, in this work, I (IsL, LiQ, LiQT, LB and ReT) and II (Gla 1, Gla 2, IsLT, LA and Lico A) drugs represented the high-release LFs and low-release LFs, respectively. More importantly, the release behaviors of all I drugs best followed the first-order equation, while the release characteristics of II drugs, except for IsLT, best fitted the zero-order equation (Table 2, Table S3). To prove the rationality of this classification, CED values were utilized to calculate the intermolecular strength of LFs-CP binary systems, and the correlation between the drug release percent in 48 h and CED values was also elucidated. Amazingly, the results were consistent with the above analysis (Figure 8d). Moreover, the correlation between the drug release percent in 48 h and the viscosity, G″, which represented the intermolecular forces of whole hydrogel systems, also verified this (Figure 4c,d). These results together confirmed the key role of intermolecular forces of drugs-CP systems in hindering drug release, especially for I drugs. Drug diffusion is also a pivotal step in drug release and is regulated by the drug concentration in the release microenvironment. For II drugs, the drug release percent in 48 h was enhanced with increasing drug solubility in PBS/PEG400 (*v*/*v*, 80/20) (Figure 5f). However, there was no correlation between I drug release percent and drug solubility in the release medium. This finding demonstrated that drug diffusion was the rate-limiting step for II drug release.

To identify the dynamic release mechanisms in different release periods, the correlations of drug release percent at different times with C=O band displacement values and CED values were analyzed, respectively. For I drugs (IsL, LiQ, LiQT, ReT and LB), a maximum release percent was obtained at 24 h. Moreover, I LF release rates almost reached their maximum values at 6 h (Figure 2). For II drugs (Gla 1, Gla 2, IsLT, LA and Lico A), drugs release from polymers was maintained at a stable rate. Therefore, 6, 12, 24, and 48 h and 12, 24, 36 and 48 h were selected for correlation analysis of I drugs and II drugs, respectively. We found that I drug release percent values at different times all possessed a negative relationship with the C=O bands’ displacement (Figure 3c–f) and CED values (Figure 8a–d), respectively, and the correlation coefficients were enhanced as the release time increased. These underscored the increasingly dominant role of intermolecular forces in hindering I drug release within 48 h. In the meantime, the drug solubility in the release medium only affected I drug release in the first 12 h, and the correlation coefficients were significantly lower than those between the intermolecular forces and I drug release percent (Figure 5a,b). For drugs with high solubility in the release medium, the hydrogel relaxation rather than drug diffusion was the main rate-limiting process, and decreasing the intermolecular forces in the hydrogel systems was an effective way to improve the drug release.

On the other hand, values for II drug release percent all showed increasing trends as drug solubility in the release medium increased at 12, 24, 36 and 48 h, in which the correlation coefficients showed an increase at 24 h followed by a significant decrease after 36 h (Figure 5c,f). This was attributed to the increasing drug concentration in receptor compartments of diffusion cells, which hindered drug release after 36 h. These findings further emphasized the decisive role of drug solubility in the release medium for II drug release, as discussed before. In fact, I drugs possessed significantly higher solubility in the release medium than II drugs, which determined whether the drug release was controlled by drug diffusion or release relaxation. Therefore, for insoluble drugs (II drugs), enhancing the drug solubility in the release medium was an effective and reasonable strategy to improve the drug release. Drug release diffusion was also regulated by the mesh size of the hydrogels. It was shown that the mesh size on the surface of I drugs-CP hydrogels was significantly larger than that of II drugs-CP hydrogels (Figure 7 and Table S1), which also served as an important indicator to distinguish the high-release and low-release LFs [17,39].

Log P of the LFs compounds was an important indicator for prediction of drug release behaviors (Figure 10). For high-release LFs compounds, log P mainly affected the compatibility between the drug and the CP matrix and hydrogel relaxation process. A lower log P value led to a lower intermolecular strength between the drug and matrix (Figure 9b,c), which was beneficial for I drug release. On the other hand, log P predominantly impacted the II drug solubility in the release medium and the drug diffusion process. A higher log P resulted in a lower drug solubility in the release medium (Figure 9a), which restricted II drug release. These results were consistent with the previous study demonstrating the negative relationship between drug release percent and log P [40].

With regard to the effect of polar surface area and polarizability on I drug release, we found that the C=O bands’ displacement values decreased as the polar surface area (R^2^ = 0.72) and polarizability (R^2^ = 0.58) increased. These findings suggested that hydrogen bond or van der Waals forces may be the driving forces that controlled I drug release. Higher polar surface area and polarizability usually requires stronger hydrogen bond or van der Waals forces to form [41]. However, the results obtained in this work were the opposite, which was on account of the presence of other dominant intermolecular forces, such as hydrophobic associations. Hydrogels often provide numerous binding sites for interactions with drugs, which also potentially allow several interactions within a single hydrogel system [42]. Hydrophobic associations in the LFs-CP hydrogels represented the intermolecular forces forming between the hydrophobic sites of LFs compounds and CP hydrogels. Previous studies revealed that the hydrophobic association forces in hydrogels increased along with increasing log P of the drugs [40,43,44], which was in accord with this study. Taken together, these findings suggest hydrophobic association forces might be the driving forces controlling the release of I LFs compounds.

To obtain more insight into the structure−release capability relationship, the release behaviors of IsL and LiQ and of LiQT and IsLT were compared, respectively. IsL and LiQ showed no significant differences in log P, polar surface area and polarizability, which led to similar C=O band displacement values and CED values; thus, a similar drug release percent was obtained. However, IsLT possessed a significantly higher log P than LiQT; therefore, LiQT was classified as high-release LFs while IsLT was classified as low-release LFs. Log P of LFs helps predict the LFs release properties from CP hydrogels.

## 5. Conclusions

In this work, we found that the log P value of LFs serves as a bridge to control the LFs release from hydrogel. High-release LFs possessed significantly lower log P and higher drug solubility in the release medium than low-release LFs. Log P mainly affected the hydrogel relaxation process for I drugs release and the drug diffusion process for II drugs release. Higher log P values of drugs resulted in higher intermolecular strength for I drugs-CP binary systems and lower solubility in the release medium for II drugs, which further hindered the drug release. For I drugs, hydrophobic association forces played an increasingly dominant role in hindering drug release with increasing release time within 48 h. However, for II drugs, higher drug solubility in the release medium facilitated drug release percent and that decisive role increased in the first 24 h, followed by a significant decline. The log P values of LFs served as important indicators for the prediction of LFs release behaviors and classification from CP cross-linked hydrogels, and the study provided references for flavonoids hydrogel design in pharmaceutical topical formulations.

## Figures and Tables

**Table 1:** The physicochemical parameters of different LFs compounds.

	Molecular Weight (M.W.)	Hydrogen Bond Donors	Hydrogen Bond Receptors	Polarizability	Log P	Polar Surface Area (Å)	Drug Solubility in PBS/PEG400 (*v*/*v*, 80/20) (μg/mL)
LiQ	418.40	5	9	40.5	0.61	146.0	421.55
IsL	418.40	6	9	42.3	0.76	157.0	414.59
LiQT	256.25	2	4	27.1	2.76	66.8	353.44
IsLT	256.25	3	4	28.8	3.11	77.8	88.17
Gla 1	324.40	2	4	36.1	4.26	58.9	121.37
LA	338.40	2	4	39.8	4.95	66.8	25.20
ReT	270.28	2	4	30.7	3.23	66.8	90.49
LB	286.28	3	5	31.5	2.57	87.0	492.57
Lico A	322.40	2	4	36.2	4.64	66.8	5.34
Gla 2	336.30	2	3	35.9	5.34	76.0	22.09

The physicochemical parameters were obtained using advanced chemistry development (ACD/Labs) software V11.02.

**Table 2:** The release equations and R^2^ calculations of 10 LFs from CP cross-linked hydrogels.

	Equation	Best Fit Order	R^2^
LiQ	Y = 102.26 × (1 − e^−0.12t^)	First order	0.95
IsL	Y = 98.45 × (1 − e^−0.14t^)	First order	0.94
LiQT	Y = 80.79 × (1 − e^−0.27t^)	First order	0.99
IsLT	Y = 9.72 × t^0.50^ − 10.03	Higuchi	0.96
Gla 1	Y = 1.58 × t + 0.68	Zero order	0.97
LA	Y = 1.02 × t + 0.22	Zero order	0.99
RET	Y = 65.71 × (1 − e^−0.079t^)	First order	0.96
LB	Y = 74.30 × (1 − e^−0.16t^)	First order	0.95
Lico A	Y = 0.17 × t + 0.18	Zero order	0.98
Gla 2	Y = 1.15 × t − 0.52	Zero order	0.99

## Data Availability

Not applicable.

## Supplementary Materials

The following supporting information can be downloaded at: https://www.mdpi.com/article/10.3390/pharmaceutics14071333/s1. Figure S1: Chemical structures of 10 LFs compounds; Figure S2: Correlation analysis between the drug release percent and C=O, –OH bands’ displacement values; Figure S3: Flow characterization of the LiQ-CP, LiQT-CP, LB-CP, IsLT-CP, LA-CP and Gla 2-CP hydrogels; Figure S4: Correlation analysis between the drug release percent and polar surface area, polarizability; Figure S5: The network structures and the pore size distributions of the LiQT-CP, LB-CP, IsLT-CP, LA-CP and Gla 2-CP hydrogels; Figure S6: Correlation analysis between drug release percent and E_mix_ values; Figure S7: Snapshots of systems at the end of the MD; Figure S8: The HPLC analysis of the ten LFs compounds; Table S1: The average pore size of the different hydrogel; Table S2: The χ and CED values of the simulated LFs-CP systems; Table S3: the release equations and R2 calculations of 10 LFs from CP cross-linked hydrogels.
