# Supplementary material for: Log P Determines Licorice Flavonoids Release Behaviors and Classification from CARBOMER Cross-Linked Hydrogel
Source: Pharmaceutics. 2022 Jun 24;14(7):1333. doi: 10.3390/pharmaceutics14071333 (PMC9322780; doi:10.3390/pharmaceutics14071333)
Supplement: Supplementary file 1 [file pharmaceutics-14-01333-s001.zip › pharmaceutics-1762225-supplementary.pdf]

Supplementary materials

Log P determines licorice flavonoids release behaviors and classification from Carbomer cross-linked hydrogel

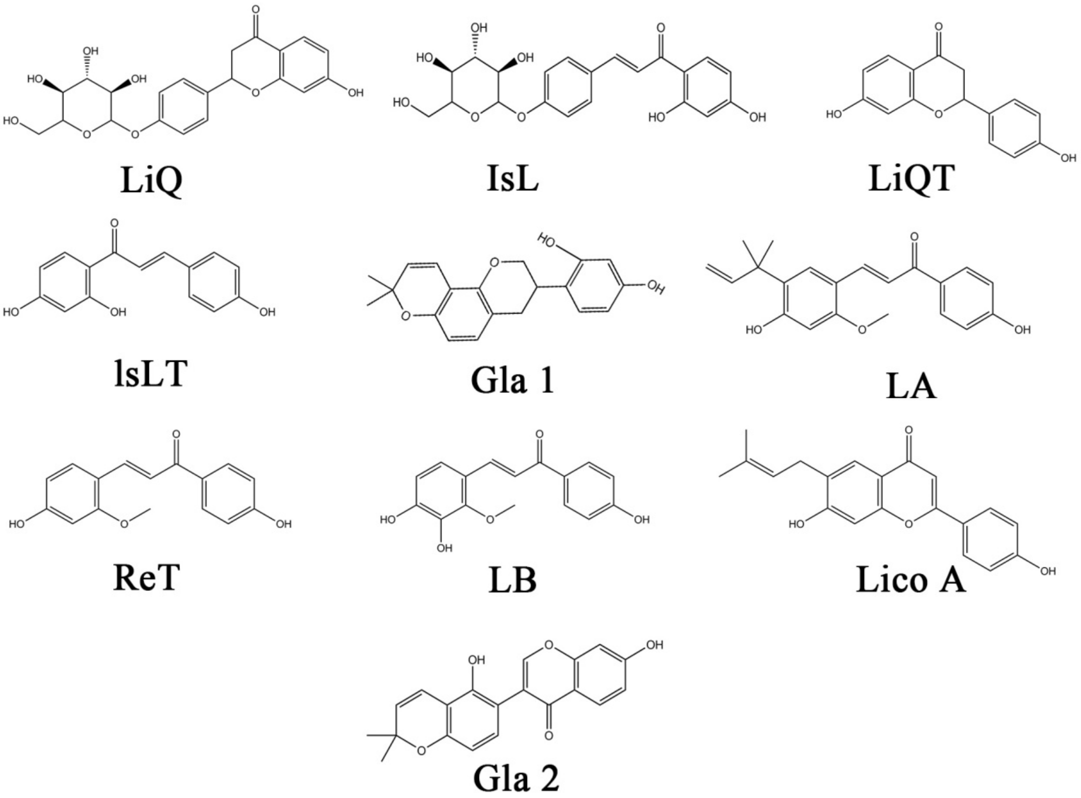

Figure S1: Chemical structures of 10 LFs compounds

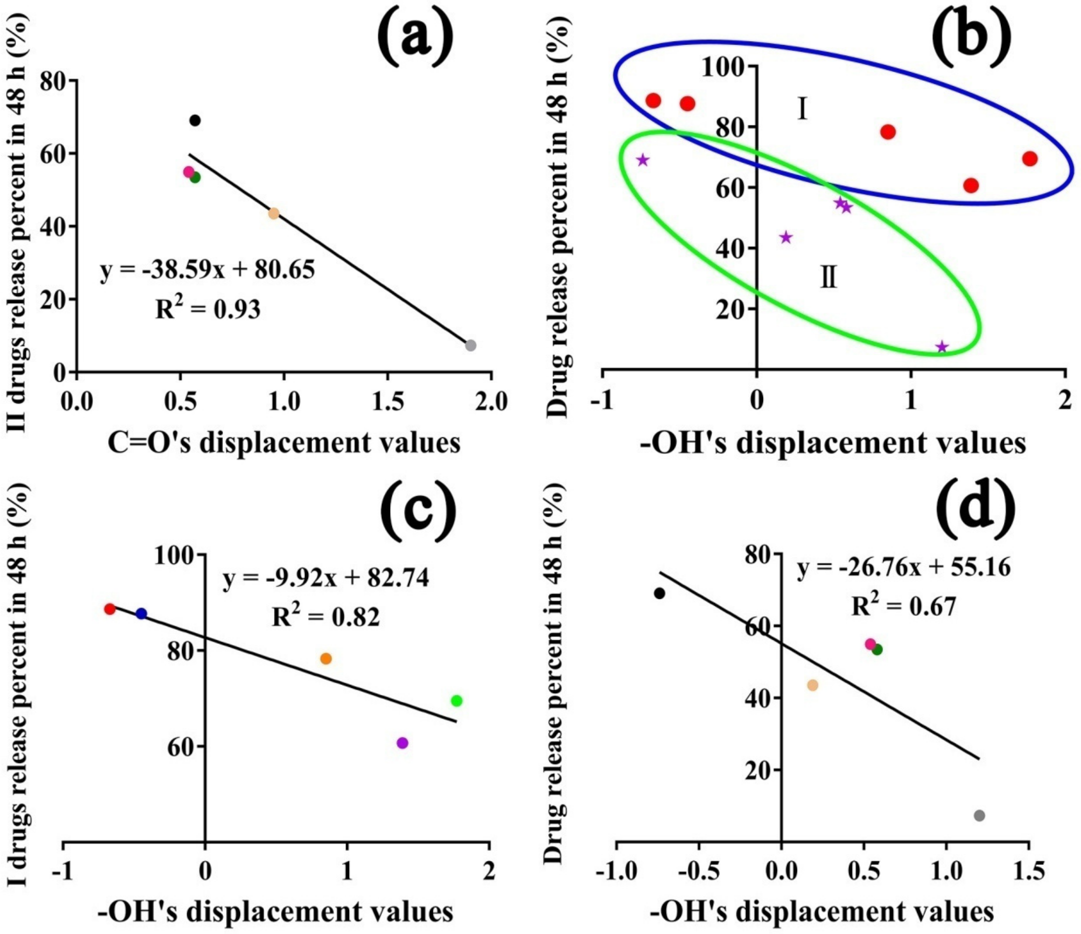

Figure S2: Correlation analysis between the drug release percent and C=O, –OH bands' displacement values (a) The relationship between II drugs (low-release LFs) release in 48 h and C=O bands' displacement values in FTIR; (b) The linear correlation of the ten kinds of LFs release percent in 48 h and –OH bands' displacement values; The relationship between I drugs (c), II drugs (d) release in 48 h and –OH bands' displacement values in FTIR.

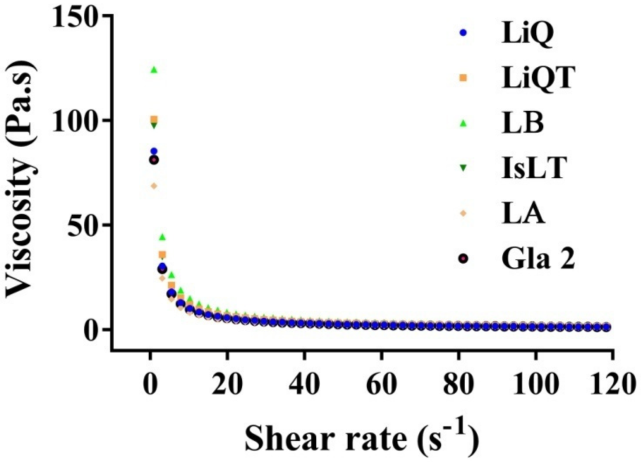

Figure S3: (a) Flow characterization of the LiQ-CP, LiQT-CP, LB-CP, IsLT-CP, LA-CP and Gla 2-CP hydrogels.

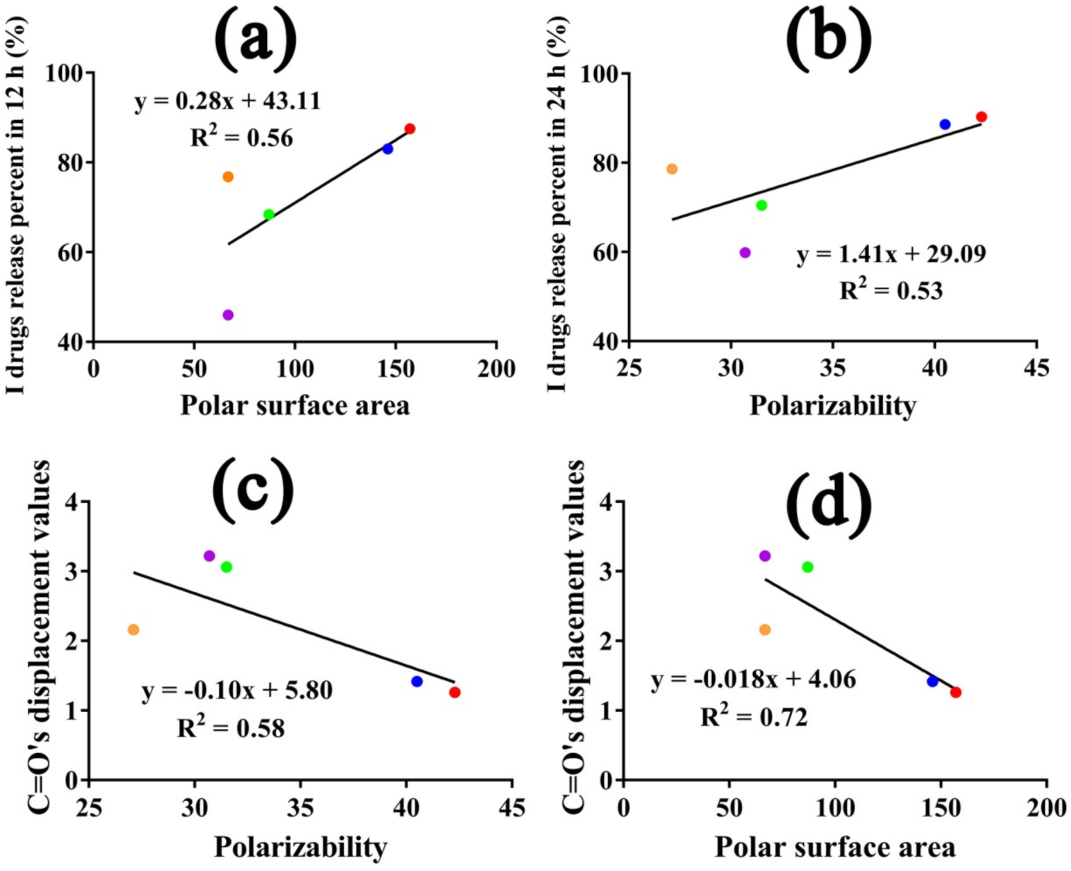

Figure S4: Correlation analysis between the drug release percent and polar surface area, polarizability. (a) The linear relationship between I drugs release percent in 12 h and polar surface area of the high-release LFs; (b) The linear correlation of I drugs release percent in 24 h polarizability; The negative correlation between C=O bands' displacement values of I drugs in FTIR analysis and polarizability (c), polar surface area (d).

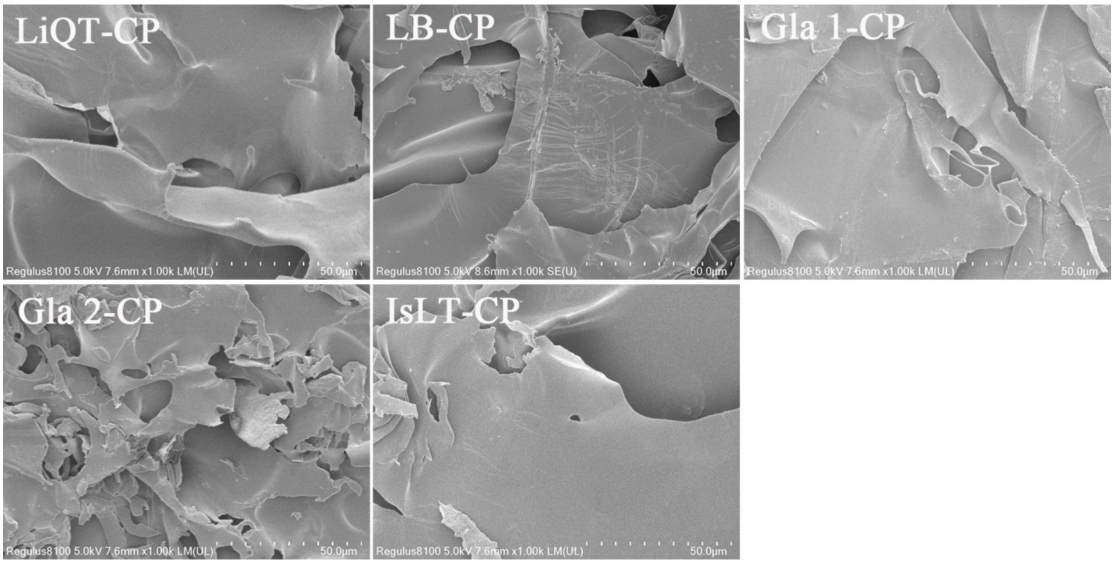

Figure S5: The network structures and the pore size distributions of the LiQT-CP, LB-CP, IsLT-CP, LA-CP and Gla 2-CP hydrogels. (Bar=50 μm)

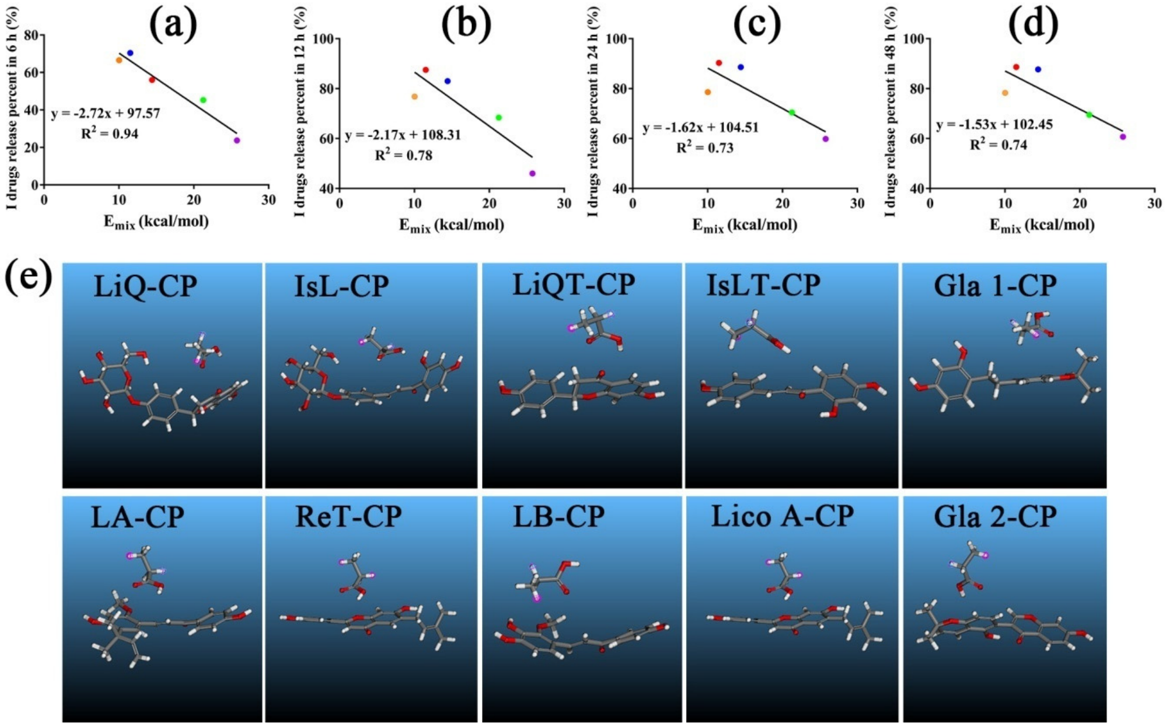

Figure S6: Correlation analysis between drug release percent and E_{mix} values. The correlation between I drugs release percent in 6 h (a), 12 h (b), 24 h (c), 48 h (d) and E_{mix} values of I drugs-CP binary systems, respectively; (e) Conformations of ten LFs-CP.

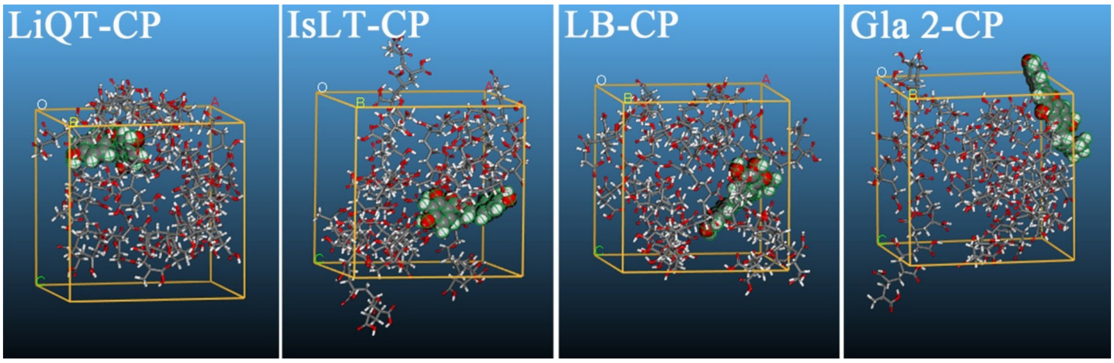

Figure S7: Snapshots of systems at the end of the MD. (LiQT-CP, LB-CP, IsLT-CP and Gla 2-CP hydrogels)

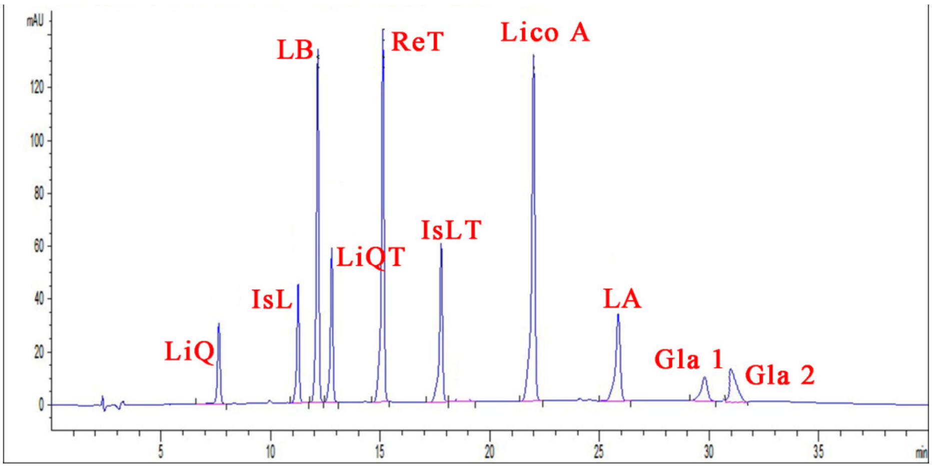

Figure S8 The HPLC analysis of the ten LFs compounds

Table S1 The average pore size of the different hydrogel

Hydrogel	Pore size (μm)
LiQ-CP	33.1±3.8
IsL-CP	30.5±2.6
LiQT-CP	28.7±4.1
IsLT-CP	14.9±2.2
Gla 1-CP	18.1±2.4
LA-CP	16.7±2.9
ReT-CP	22.1±2.8
LB-CP	26.3±1.9
Lico A-CP	4.6±0.5
Gla 2-CP	12.1±1.9
Blank CP	27.4±3.8

Table S2 The χ and CED values of the simulated LFs-CP systems

	χ (Kcal/mol)	CED ($1*10^9$, Kcal/mol)
LiQ-CP	14.43	2.08
IsL-CP	11.50	2.08
LiQT-CP	10.02	2.18
IsLT-CP	30.64	2.34
Gla 1-CP	13.00	2.33
LA-CP	20.52	2.59
RET-CP	25.75	2.51
LB-CP	21.26	2.40
Lico A-CP	32.35	2.25
Gla 2-CP	25.84	2.62

Table S3 The release equations and R^2 calculations of 10 LFs from CP cross-linked hydrogels

		Equation	R^2
LiQ	Zero order	$Y=4.38\times t + 12.25$	0.72
	First order	$Y=102.26\times(1-e^{-0.12t})$	0.95
	Higuchi	$Y=25.51\times t^{0.50}-15.57$	0.89
IsL	Zero order	$Y=4.22\times t + 15.11$	0.67
	First order	$Y=98.45\times(1-e^{-0.14t})$	0.94
	Higuchi	$Y=24.88\times t^{0.50}-12.36$	0.86
LiQT	Zero order	$Y=3.28\times t + 26.25$	0.54
	First order	$Y=80.79\times(1-e^{-0.27t})$	0.99
	Higuchi	$Y=20.59\times t^{0.50}+2.14$	0.80
IsLT	Zero order	$Y=1.27\times t + 2.62$	0.90
	First order	-	-
	Higuchi	$Y=9.72\times t^{0.50}-10.03$	0.96
Gla 1	Zero order	$Y=1.02\times t + 0.22$	0.97
	First order	$Y=111.55\times(1-e^{-0.021t})$	0.95
	Higuchi	$Y=12.08\times t^{0.50}- 14.55$	0.96
LA	Zero order	$Y=1.02\times t + 0.22$	0.99
	First order	-	-
	Higuchi	$Y=7.42\times t^{0.50}- 14.55$	0.96
ReT	Zero order	$Y=1.43\times t + 9.37$	0.73
	First order	$Y=65.71\times(1-e^{-0.079t})$	0.96
	Higuchi	$Y=11.63\times t^{0.50}-6.80$	0.89
LB	Zero order	$Y=1.43\times t + 23.25$	0.46
	First order	$Y=74.30\times(1-e^{-0.16t})$	0.95
	Higuchi	$Y=12.75\times t^{0.50}+ 3.86$	0.71
Lico A	Zero order	$Y=0.17\times t + 0.18$	0.98
	First order	$Y=10.52\times(1-e^{-0.025t})$	0.97
	Higuchi	$Y=1.24\times t^{0.50}- 1.40$	0.97
Gla 2	Zero order	$Y=1.15\times t - 0.52$	0.99
	First order	-	-
	Higuchi	$Y=8.31\times t^{0.50}- 10.57$	0.93

- represented that the equation cannot be fitted.
